# Post-coronavirus disease 2019 polyneuropathy with significant response to immunoglobulin therapy: a case report

**DOI:** 10.1186/s13256-021-03148-y

**Published:** 2021-11-02

**Authors:** A. Saleh, R. Jung, S. Tonner, F. Hornof, M. Strittmatter

**Affiliations:** 1 Neurology Department, SHG Klinikum Merzig, 66663 Merzig, Germany; 2 Neurology Department, Medizinisches Versorgungszentrum Merzig, 66663 Merzig, Germany

**Keywords:** COVID-19, Neuropathy, Post-COVID-19 syndrome, Case report

## Abstract

**Background:**

The symptoms of coronavirus disease 2019, caused by the novel severe acute respiratory syndrome coronavirus 2, were originally assumed to be mainly respiratory. With increasing knowledge, however, it turned out that the spectrum of complaints varies broadly with age and concomitant diseases. While many neurological symptoms were reported in the context of the disease, ranging from permanent fatigue to recurrent headaches and concentration disturbance, treatment approaches are still in development. This case discusses a possible treatment approach with immunoglobulin therapy and its outcomes.

**Case presentation:**

We present the case of a 56-year-old Caucasian female patient who, following coronavirus disease 2019, developed peripheral sensory and autonomic disturbances that fell within subacute demyelinating neuropathy. Because a significant improvement in symptoms as well as in the results of clinical and electrophysiological examination was reported after immunoglobulin therapy, long-term therapy does not appear to be necessary.

**Conclusion:**

Given the significant subjective and objective improvement reported, this case provides additional evidence that immunoglobulin therapy can be considered in post-coronavirus disease 2019 syndrome.

## Background

The disease caused by the novel coronavirus severe acute respiratory syndrome coronavirus 2 (SARS-CoV-2) is known as coronavirus disease 2019 (COVID-19). The symptoms were originally assumed to be mainly respiratory. With increasing knowledge, however, it turned out that the spectrum of complaints varies broadly with age and concomitant diseases. Mild courses, similar to a cold, up to severe disease with fatal outcomes are possible. While many nonrespiratory symptoms were mentioned in the context of the disease [1], neurological symptoms reported range from permanent fatigue, recurrent headaches, and concentration disturbance to polyneuropathy-related complaints [2]. This case reports various neurological symptoms in the postinfectious phase that are mostly attributed to an autoimmune mechanism [3] and discusses the use of immunoglobulin therapy as a treatment approach.

## Case presentation

A 56-year-old Caucasian female patient presented with polyneuropathy-related complaints that manifested directly after a quarantine for a COVID-19 infection. At the time of admission, about a month after contracting the disease, the patient showed a mild viral load that was attributed to dead viral remnants. Although the previously reported respiratory symptoms of the disease had regressed, the patient reported painful abnormal sensations on the hands and feet with symmetrical acral distribution, which she described as the sensation of cold water and “1000 pinpricks.” In the course of a few days, a changed sense of touch that affected the patient’s gait was noticed distally on the legs. She felt insecure climbing stairs, with no definite paresis but rather a general weakness. No metabolic diseases were reported other than substituted hypothyroidism.

On clinical examination, the patient was alert and fully oriented with normal cranial nerves. No signs of ataxia or paresis were found other than a positive Phalen test. Muscle reflexes of the upper extremities as well as the patellar reflex were exaggerated, while the Achilles reflex was weak on both sides with no pyramidal signs. Distally symmetrical hypoesthesia on the hands and feet as well as pallhypesthesia on the metatarsophalangeal joint and the malleolus medialis on both sides was reported.

## Investigations

Electrophysiological examination of peripheral nerves revealed demyelinating changes consistent with acute/chronic inflammatory demyelinating polyneuropathy given the course of time (less than 8 weeks at the time). Nerve ultrasound showed thickened nerve roots and corroborated the suspicion of immune-mediated genesis of the polyneuropathy. Involvement of the autonomic nervous system was also detected using autonomic testing.

For further investigation, a lumbar puncture was carried out and showed a discrete albuminocytological dissociation that supported the suspected diagnosis. This was followed by magnetic resonance imaging of the cervical spine to clarify the broadened reflexes. However, no spinal cord affection was found.

## Treatment

With a normal level of IgA, a therapeutic attempt was carried out with a dose of 0.4 g/kg body weight of immunoglobulin on five consecutive days with thrombosis prophylaxis using 40 mg enoxaparin sodium and rheological therapy. Apart from a slight headache, no side effects were reported. Already during the therapy, the patient noticed a slight improvement in the symptoms at rest. However, the symptoms flared up again with exertion.

## Outcome and follow-up

**Fig. 1:**
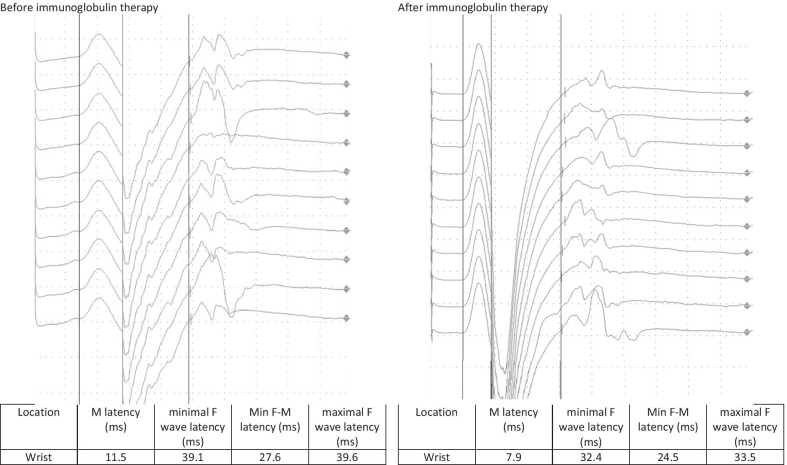
F-wave of the right median nerve before and after immunoglobulin therapy

**Fig. 2:**
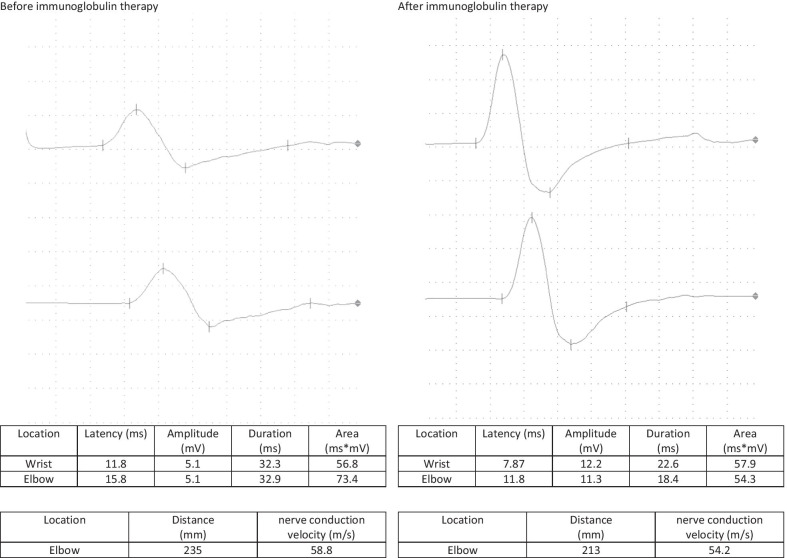
Nerve conduction velocity of the right median nerve before and after immunoglobulin therapy

**Fig. 3:**
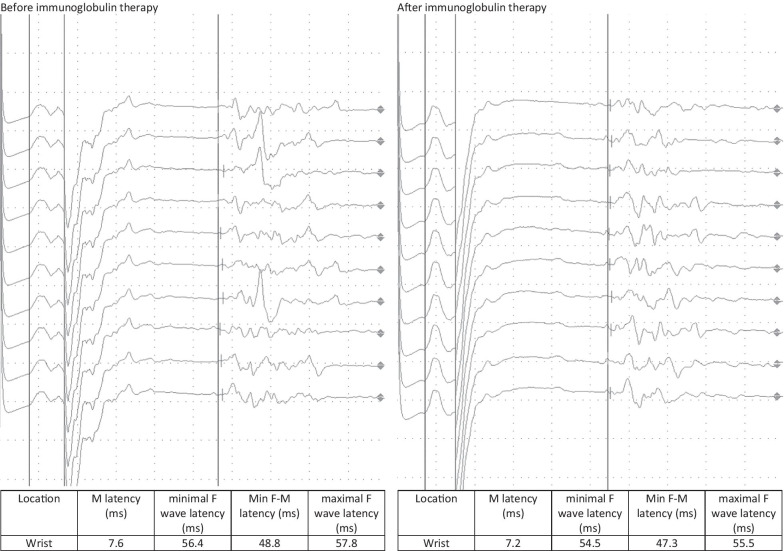
F-wave of the right tibial nerve before and after immunoglobulin therapy

**Fig. 4:**
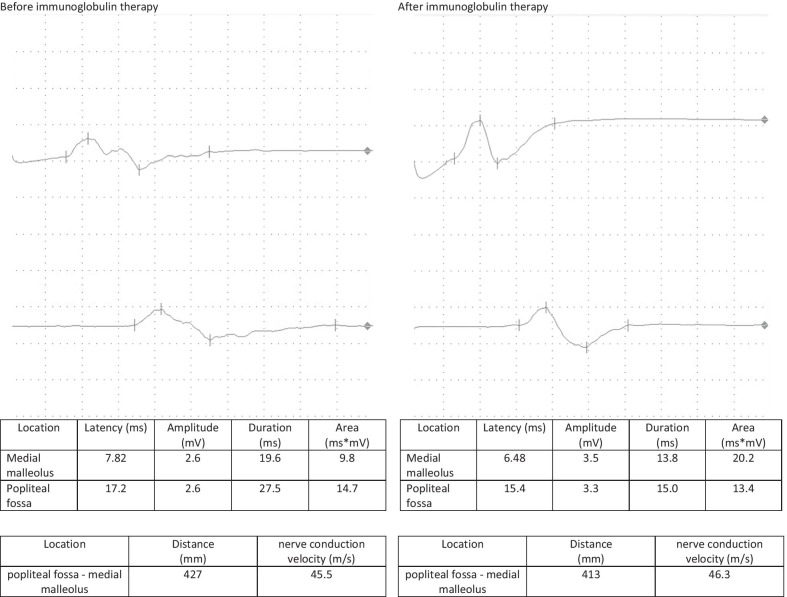
Nerve conduction velocity of the right tibial nerve before and after immunoglobulin therapy

A follow-up appointment for clinical and electrophysiological follow-up was carried out after 4 weeks. The patient reported a headache for 5 days after the administration of the immunoglobulin therapy followed by a significant improvement in the complaints, especially with regard to the hypoesthesia and gait, while only minimal residual hypoesthesia at the tips of fingers, toes, and the nose persisted. The sensorimotor demyelinating polyneuropathy continued to improve on electrophysiological examination, with the electrophysiological criteria of chronic inflammatory demyelinating polyneuropathy no longer being fulfilled. A second dose of 90 g of immunoglobulin was administered without any complications (Figs. 1, 2, 3, 4).

## Discussion

This report presents a case of a peripheral sensory and autonomic disturbance with demyelinating changes following COVID-19 disease. The symptoms as well as the electrophysiological changes were attributed to a postinfectious immune-mediated process [4], which was recently reported in COVID-19 patients with varying symptoms [1]. However, the exact mechanism of this autoimmune reaction as a trigger of this phenomenon [3] as well as the mechanism of the sensory disturbances themselves, in terms of being a neurofibrillary disorder or an acute/chronic inflammatory myeloid disorder, requires more research [4, 5].

Although the symptoms showed significant regression after the immunoglobulin therapy, it was not possible in the absence of clear guidelines to decide whether to continue the immunoglobulin therapy. This was left to be decided in light of the clinical findings in the course of the disease. At the time, long-term immunoglobulin therapy did not appear to be necessary.

## Conclusion

This case provides additional evidence that immunoglobulin therapy can to be considered in post-COVID-19 syndrome given the significant subjective and objective improvement reported. In this regard, a good prognosis is to be expected.

## Learning points/take-home messages

- Chronic neurological symptoms are being frequently reported in the context of post-COVID-19 syndrome.
- Sensory polyneuropathy can occur in the postinfectious phase and is attributed to an immune-mediated processes. Immunoglobulin therapy can be considered as a treatment option with an expected good prognosis.

## Patient’s perspective

The COVID-19-disease and the quarantine were for me, on the personal as well as on the family level, a bitter experience. It was however more shocking when the neurological symptoms occurred after a while and I was told of their relationship to the disease, so that it seemed possible at that time that the symptoms would persist and I would always have something to remind me of this experience. Everything was new for me as well as for the physicians treating me. Every question, especially regarding the long-term treatment, needed time and further reading.

The first round of the immunotherapy went slow, while I barely noticed anything other than a headache. I though at the beginning while laying down in my hospital bed that me symptoms were getting better. However, they proved my wrong with the slightest effort I did.

The same continued after discharge. However, five days later I started noticing something. The painful numbness on my hands and toes as well as on my nose started to take a turn one day after the other. I could also walk better. Today, a month later I only have minimal numbness on the tip of my nose, which I hope it would go also away with time.

## Data Availability

The datasets used and/or analyzed during the current study are available from the corresponding author on reasonable request.
